# Advances in Quantitative Analysis of ^18^F-Sodium Fluoride Coronary Imaging

**DOI:** 10.1155/2021/8849429

**Published:** 2021-01-15

**Authors:** Jacek Kwiecinski, Martin Lyngby Lassen, Piotr J. Slomka

**Affiliations:** ^1^Department of Imaging (Division of Nuclear Medicine), Medicine, and Biomedical Sciences, Cedars-Sinai Medical Center, Los Angeles, CA, USA; ^2^Department of Interventional Cardiology and Angiology, Institute of Cardiology, Warsaw, Poland

## Abstract

^18^F-sodium fluoride (^18^F-NaF) positron emission tomography (PET) has emerged as a promising noninvasive imaging tool for the assessment of active calcification processes in coronary artery disease. ^18^F-NaF uptake colocalizes to high-risk and ruptured atherosclerotic plaques. Most recently, ^18^F-NaF coronary uptake was shown to be a robust and independent predictor of myocardial infarction in patients with advanced coronary artery disease. In this review, we provide an overview of the advances in coronary ^18^F-NaF imaging. In particular, we discuss the recently developed and validated motion correction techniques which address heart contractions, tidal breathing, and patient repositioning during the prolonged PET acquisitions. Additionally, we discuss a novel quantification approach—the coronary microcalcification activity (which has been inspired by the widely employed method in oncology total active tumor volume measurement). This new method provides a single number encompassing ^18^F-NaF activity within the entire coronary vasculature rather than just information regarding a single area of most intense tracer uptake.

## 1. Introduction

(^18^F-NaF) is an established positron emission tomography (PET) bone tracer that binds to hydroxyapatite, a crystalline structure that is a key component of bones which also is present in atherosclerotic plaques [1, 2]. Aside from being a hallmark of the disease, vascular calcifications can be quantified and used to characterize the extent of the disease burden [3, 4]. In addition to standardizing the assessments, the quantification of the extent and volume of calcified atherosclerotic plaques conveys prognostic information and is routinely employed for risk stratification [5–7]. While vascular macrocalcifications as identified on computed tomography (CT) imaging represent an end-stage stable condition of coronary plaque progression and maturation, the active calcification process (microcalcification) is associated with atherosclerotic plaque progression that might turn into either vulnerable plaques (unstable disease) [8, 9] or macrocalcifications which can be visualized on noninvasive computed tomography imaging [10–13].

## 2. Initial ^18^F-NaF Cardiovascular Imaging

**Figure 1:**
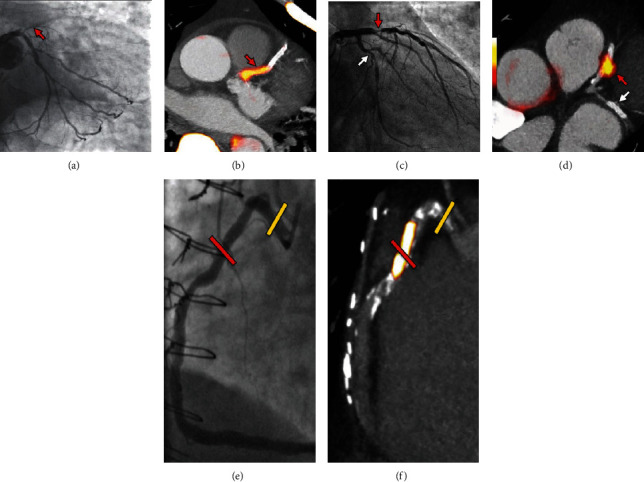
Focal ^18^F-NaF in patients with myocardial infarction and stable angina. A patient with acute ST-segment elevation myocardial infarction with (a) proximal occlusion (red arrow) of the left anterior descending artery on invasive coronary angiography and (b) intense focal ^18^F-NaF (tissue-to-background ratios, culprit 2.27 vs. reference segment 1.09 (108% increase)) uptake (yellow-red) at the site of the culprit plaque (red arrow) on the combined PET and CT. A patient with anterior non-ST-segment elevation MI with (c) culprit (red arrow; left anterior descending artery) and bystander nonculprit (white arrow; circumflex artery) lesions on invasive coronary angiography that were both stented during the index admission. Only the culprit lesion had increased ^18^F-NaF uptake (tissue-to-background ratios, culprit 2.03 vs. reference segment 1.08 (88% increase)) on PET-CT (d) after percutaneous coronary intervention. In a patient with stable angina with previous coronary artery bypass grafting, (e) invasive coronary angiography showed nonobstructive disease in the right coronary artery. (f) Corresponding PET-CT scan showed a region of increased ^18^F-NaF activity (positive lesion, red line) in the midright coronary artery (tissue-to-background ratio, 3.13) and a region without increased uptake in the proximal vessel (negative lesion, yellow line). This figure was originally published in the *Lancet* under the Creative Commons Attribution 4.0 International License (http://creativecommons.org/licenses/by/4.0/) (data from Joshi et al. [20]).

^18^F-sodium fluoride has been used in oncological assessments of bone cancer and metastases for nearly 5 decades [1, 14]. Over a decade ago, retrospective studies in patients evaluated for oncological purposes utilizing ^18^F-NaF PET found that many patients also had ^18^F-NaF uptake in the cardiovascular system [15–18]. Derlin et al. observed ^18^F-NaF uptake within the aorta in patients with and without visual identifiable calcification in the aortic wall on CT images. Analogous findings were subsequently reported by other groups [16–18]. These initial studies sparked interest in ^18^F-NaF cardiovascular imaging as it was hypothesized that ^18^F-NaF uptake might be an early marker of atherosclerosis preceding macrocalcifications. Dweck et al. showed that while there is a strong correlation between ^18^F-NaF uptake and the coronary calcium score (a direct measure of the coronary artery disease burden), over 40% of patients with exceptionally high coronary calcium scores (>1000) show no PET tracer uptake [19]. The study suggested that ^18^F-NaF coronary uptake provides complementary information compared to measures of coronary macrocalcification as it correlates with prior major adverse cardiovascular events, patient symptoms, and risk scores. The same group went on to show that ^18^F-NaF PET/CT is the first noninvasive imaging method to identify and localize ruptured and high-risk coronary plaques (Figure 1) [20]. On histological examinations, ^18^F-NaF activity has been observed in regions of plaque rupture with evidence of increased inflammation, calcification activity, necrosis, and cell death. These promising findings lead to the commencement of large observational studies aimed at exploring the prognostic implications of ^18^F-NaF coronary PET imaging and interventional studies assessing the potential of this imaging modality for guiding treatment (Prediction of Recurrent Events With ^18^F-Fluoride (PREFFIR) NCT02278211, Dual Antiplatelet Therapy to Reduce Myocardial Injury (DIAMOND) NCT02110303, and Effect of Evolocumab on Coronary Artery Plaque Volume and Composition by CCTA and Microcalcification by ^18^F-NaF PET (EVOLVE) NCT03689946 [21–23]).

## 3. Current Methods of Measuring ^18^F-NaF Uptake and Their Limitations

Two different quantification techniques of coronary ^18^F-NaF uptake have been proposed, one evaluating the whole-heart uptake (global coronary method) and another focusing on the uptake within the coronary arteries (coronary-specific method). The global coronary method employs a whole-heart segmentation, with manual delineation of the cardiac silhouette on consecutive axial slices of fused PET/CT images [24]. This method of coronary analysis was first described by Beheshti et al. in a retrospective study of oncological patients [25]. The global ^18^F-NaF activity can be calculated using a volume-corrected summation of the mean standardized uptake value (SUVmean) of each of the regions of interest drawn on consecutive images. A different approach evaluating the uptake in individual coronary plaques (coronary-specific method) was proposed by Dweck et al. and later refined by Joshi et al. [19, 20]. This technique relies on the identification of coronary lesions with focal ^18^F-NaF uptake, employing coronary CT angiography to visually identify atherosclerotic plaques. Regardless of the method utilized for ^18^F-NaF assessment, the detected radiotracer activity in an atherosclerotic plaque is affected by the blood pool activity [2, 26]. To offset this phenomenon rather than reporting crude maximum standardized uptake values (SUVmax), researchers have adjusted for blood pool ^18^F-NaF activity, providing a maximum target-to-background (TBRmax) output (TBRmax = maximum SUV of the lesion/mean SUV obtained in the background blood pool). The calculation of the TBRmax, however, is affected by the changes in the background blood pool activity which has been shown to change over a 2-hour period ranging from 1-hour postinjection to 3-hour postinjection while coronary lesion uptake remains consistent [27]. To correct for variations in injection-to-scan delays, Lassen et al. have proposed to use a background blood pool correction factor that improves test-retest reproducibility measures of TBR in patient cohorts where small variations in injection-to-scan delays are possible [28].

Both the global and coronary-specific approaches have their limitations. While the global coronary method is attractive due to its simplicity, the specificity of the method is limited. This is largely due to the small coronary lesion size (which results in low plaque activity) and the fact that it is prone to false-positive findings due to myocardial, valvular, or pericardial uptake as well as background noise [29]. The global cardiac uptake method relies on the assumption that any cardiac ^18^F-NaF activity detected as higher than the blood pool originates from the coronary vasculature. However, there is a growing body of evidence that cardiac valves and great vessels bind ^18^F-NaF and, thus, have an impact on the accuracy of the quantitative assessments when using the global heart assessment technique [30–35]. Additionally, increased myocardial uptake of ^18^F-NaF is often observed in the infarcted myocardium, myocardial scar tissue, and cardiac amyloidosis [36–38]. All of these may falsely elevate the global ^18^F-NaF reading leading to overestimation of coronary ^18^F-NaF activity [24]. The plaque-specific approach is not prone to these caveats; however, it has limitations relating to the labor-intensive subjective assessments. The plaque-specific technique requires manual delineations of the lesions, where the reader must read the coronary tree to identify potential lesions. Here, partial volume effects and discordance between PET uptake and coronary CT angiography increase the time spent on the analyses and introduce some degree of reader dependency although recent studies have shown high interreader agreement [39]. Additionally, according to Joshi et al., the difference in the ^18^F-NaF maximum TBR in culprit compared with nonculprit plaques is only 34% which is severalfold lower than PET tracer uptake reported in oncological settings [20]. Finally, by reporting only a single highest TBR value, the overall coronary activity cannot be fully appreciated; as a result in individuals with multiple foci of uptake in their coronary vasculature, this approach can be misleading.

For precise and reproducible ^18^F-NaF coronary uptake assessments, it is essential to take full advantage of state-of-the-art image reconstruction approaches [40]. As demonstrated previously, the number of iterations and the use of filtering, as well as applying point spread functions and employing time of flight information and resolution recovery techniques, all have a profound impact on the uptake values [41]. In a recent study, Doris et al. showed how heavily the signal-to-noise ratio and image quality depend on particular reconstruction settings [42]. According to their study, while more iterations and no postfiltering result in higher uptake values, such images are excessively noisy and difficult to interpret. It is therefore essential for multicenter studies to harmonize imaging protocols. Undoubtedly, standardization of reconstruction parameters shall be an important step in disseminating ^18^F-NaF PET as it will enable making direct comparisons between centers/vendors and facilitate establishing large registry datasets [43].

## 4. Impact of Motion during the Scans of ^18^F-NaF Cardiovascular Imaging

Imaging of the cardiovascular system is associated with significant motion during the often-lengthy imaging sessions—with acquisition times extending for up to 30 minutes [20, 34, 44]. The long acquisitions pose the risks of patient repositioning events in addition to cardiac contractions and respiration (cardiorespiratory motion) [45]. A study evaluating the frequency and the impact of patient repositioning revealed that repositioning events may account for motions of up to 16 mm during the scans [46], with consequential reductions in the quantitative measures (TBR). Previous studies have shown that cardiac contractions may account for translations in the right coronary artery of 26 mm [45], and the aortic valve may move by 12 mm [47]. Respiratory translations might account for translations of 20-40 mm [48], which combined with the cardiac contractions and patient repositioning events introduce a complex motion pattern [28]. Correcting for the three motion patterns simultaneously has been shown to improve the test-retest reproducibility in studies of coronary plaques, in addition to ensuring unanimous assessments of the coronary plaques in comparison to the often-used end-diastolic reconstruction protocols [28]. In addition, preliminary results for studies on aortic valve stenosis have revealed significant increases in the TBR assessments, as well as significant reductions in the noise in the images [49]. While significant improvements in the TBR and test-retest reproducibility measures have been reported when employing the complex triple motion correction techniques, cardiac motion correction alone improves the image quality and reduces the noise in the images significantly [28]. This has been shown in studies of coronary plaques either using the reconstructed PET images [50] or during the image reconstruction through registration of the emission data using elastic registrations [51] and in studies of the aortic valves [30].

## 5. Evolving Understanding of Coronary Artery Disease: Shifting from the Vulnerable Plaque to the Vulnerable Patient and the Role of ^18^F-NaF PET

Since coronary ^18^F-NaF imaging is being considered a potential noninvasive tool for imaging and identification of high-risk coronary artery disease, the uptake quantification method should ultimately be in line with our current understanding of the coronary atherosclerotic disease. While for decades physicians and researchers have pursued the quest to identify vulnerable atherosclerotic plaques, despite major advancements in imaging technology that allow visualization of rupture-prone plaques, this approach did not translate into an improved prediction of adverse events [52]. It has been demonstrated that the degree of coronary stenosis does not predict myocardial infarction in several multicenter trials. In the PROMISE (Prospective Multicenter Imaging Study for Evaluation of Chest Pain) trial, the vast majority (77%) of cardiovascular deaths and myocardial infarctions occurred in patients with <50% lumen stenosis at baseline [53]. Similarly, in the ICONIC (Incident Coronary Syndromes Identified by Computed Tomography) study, most acute coronary syndromes were found to arise from nonobstructive lesions [54]. Further evidence questioning the rationale for the per plaque approach came from the PROSPECT trial (A Prospective Natural-History Study of Coronary Atherosclerosis) which investigated whether the detection of thin-capped fibroatheroma by virtual histology intravascular ultrasound would predict adverse clinical events [55]. In this study, 595 thin-capped fibroatheromas (which were believed to be prone to rupture and hence responsible for acute myocardial infarction) were identified in 697 patients; however, after a median follow-up of 3.4 years, only 6 lesions ruptured, with consequential myocardial infarction. Given the aforementioned studies, the evaluation and management of patients with coronary heart disease are in transition [42]. A comprehensive approach to patients with coronary heart disease is replacing a lesion-focused strategy [56]. The reason for the poor performance of the measures of plaque morphology, stenosis grade, and risk scores based on clinical characteristics in the prediction of adverse events is likely due to the fact that these approaches neglect the importance of coronary artery disease activity [57]. It was recently argued that plaque progression (which is a robust marker of adverse prognosis) should be used for patient risk stratification [58, 59]. Importantly, as shown by Doris et al., rather than being confined to serial imaging in order to predict plaque progression, baseline coronary ^18^F-NaF uptake can be utilized [60].

## 6. Coronary Microcalcification Activity

In view of the shift from a per lesion to a more global per patient approach, it is immediately apparent that for ^18^F-NaF coronary imaging, reporting only maximum TBR values for a given patient can be suboptimal. To adjust ^18^F-NaF coronary uptake quantification to the new per patient (rather than per plaque) paradigm, we recently proposed a novel semiautomated approach for image analysis [61]. The coronary microcalcification activity (CMA) characterizes ^18^F-NaF uptake throughout the entire coronary vasculature using CT angiography-derived centerlines to build 3-dimensional tubular volumes of interest around each of the main epicardial coronary arteries. Extending from the coronary-specific method, the coronary microcalcification activity method permits the evaluation of coronary ^18^F-NaF activity on a per vessel and per patient basis, providing more global assessments of disease activity in the coronary arteries than the per lesion approach.

**Figure 2:**
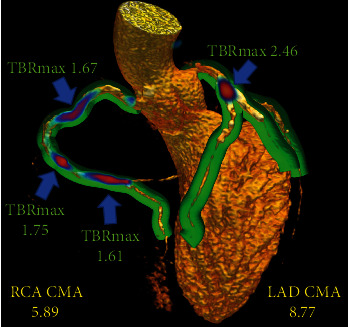
3-dimensional rendering of coronary CT angiography with superimposed tubular whole-vessel volumes of interest (light green) employed for evaluation of ^18^F-sodium fluoride uptake (blue and red). Despite the relatively lower tissue-to-background ratio maximum (TBRmax) due to multiple foci of increased ^18^F-NaF activity, the coronary microcalcification activity (CMA) in the right coronary artery (RCA) is only moderately lower than that in the left anterior descending (LAD) coronary artery which presented with a very high TBRmax. Reprinted by permission from Springer Nature: EJNMMI (Kwiecinski et al. [61]).

The coronary ^18^F-NaF uptake measure resembles the Agatston method for quantifying coronary CT calcium scores and is based on PET quantification techniques widely employed in oncology and cardiac sarcoidosis [62–64]. Tortuous 3D volumes of interest which encompass all the main epicardial coronary vessels and their immediate surroundings (4 mm radius) are automatically extracted from CT angiography datasets (Figure 2). Within such volumes of interest, the coronary microcalcification activity (CMA) representing the overall disease activity in the vessel and based upon both volume and intensity of ^18^F-NaF PET activity can be calculated as the integrated activity in SUV units exceeding the background blood pool SUVmean + 2 standard deviations. As a result, in patients and vessels with multiple foci of uptake, CMA provides a measure of disease activity across the coronary vasculature.

## 7. CMA Is Associated with Established CT Angiography-Derived Markers of Plaque Vulnerability

**Figure 3:**
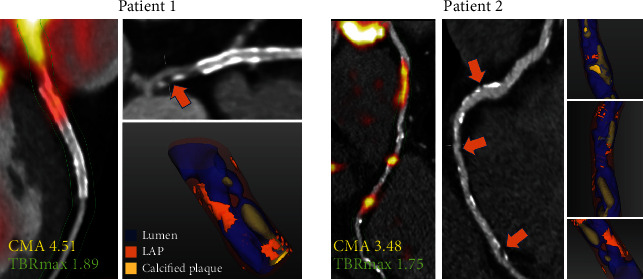
Case examples of coronary ^18^F-sodium fluoride uptake. Low attenuation plaque (LAP) on CT angiography and LAP (orange arrows) on 3D CT rendering. Right (Patient 1) and left anterior descending (Patient 2) coronary arteries. Areas of LAP correspond with areas of increased ^18^F-NaF activity. While maximum target-to-background ratio (TBRmax) reflects single hotspot activity, coronary microcalcification activity (CMA) values represent the whole-vessel ^18^F-sodium fluoride burden. Reprinted by permission from Springer Nature: EJNMMI (Kwiecinski et al. [61]).

Initial validation of the coronary microcalcification activity was carried out on a cohort of 50 individuals who underwent ^18^F-NaF PET within 3 weeks from an acute myocardial infarction. Previous studies suggested that ^18^F-NaF activity is associated with the presence and correlated with the volume of CT-derived markers of plaque vulnerability [65, 66]. Given these associations, low attenuation plaque (<30 Hounsfield units within a coronary lesion) was employed for validation of CMA [67, 68]. In the analysis, CMA outperformed the single-lesion (hotspot) TBRmax approach for the detection of low attenuation plaque [61]. Similarly, there was a stronger correlation between low attenuation plaque and CMA than between low attenuation plaque and conventional measurements of ^18^F-NaF activity with better categorical separation (Figure 3). Given that low attenuation plaque has repeatedly been shown to be associated with adverse outcomes, the discrepancy in the correlations and diagnostic accuracy between CMA and TBR/SUV supports the view that in characterizing disease severity, CMA is superior to traditional uptake measures [67, 68].

## 8. CMA Is a Reproducible and Highly Repeatable ^18^F-NaF Uptake Measure

For an imaging biomarker to have clinical utility, it should demonstrate precise and reproducible analytical performance [69, 70]. Multiple studies have demonstrated how an approach that combines visual discrimination with quantitative TBR measurements enables reliable characterization of ^18^F-NaF localization at the level of the coronary plaque with high levels of agreement between observers and across serial scans [19, 28, 70]. As discussed above, reproducibility and repeatability of SUV-based uptake measurements benefit from motion correction techniques [28, 50]. We have recently evaluated the intra- and interobserver repeatability and interscan reproducibility of the coronary microcalcification activity [39]. By leveraging the imaging data of 20 patients who underwent ^18^F-NaF PET twice within 2 weeks, we were able to show that both the TBR and CMA assessments are repeatable and reproducible, albeit CMA has superior repeatability and reproducibility. Furthermore, in our study, there was no diagnostic discordance between observers and scans for CMA, something that is not always true for TBR. Indeed, for the presence of ^18^F-NaF activity (CMA > 0) or absence of activity (CMA = 0) in the coronary tree, there was 100% agreement in the categorization between observers and scans. This is clinically relevant especially in populations without known coronary artery disease if ^18^F-NaF is to be used as a screening tool where little or no activity is expected. In such a population, the fact that CMA = 0 is perfectly reproducible between observers and scans is of extreme importance as it could reliably inform the physician regarding the coronary microcalcification activity in a given patient.

## 9. CMA Predicts Outcomes in Patients with Established Coronary Artery Disease

**Figure 4:**
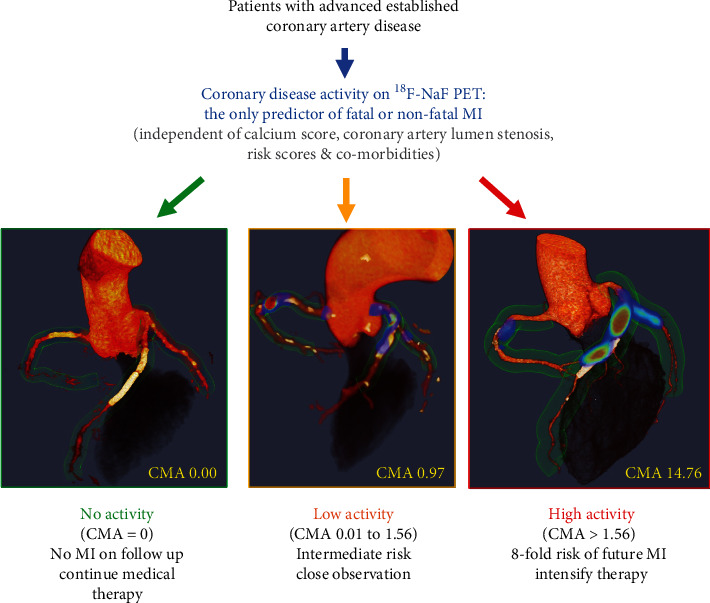
^18^F-sodium fluoride positron emission tomography as a marker of disease activity in the coronary arteries is a predictor of fatal or nonfatal myocardial infarction (MI) in patients with established coronary artery disease. ^18^F-NaF PET can be used to measure disease activity across the coronary vasculature and to stratify patients into those with no, low, and high disease activity. Patients with high disease activity (coronary microcalcification activity (CMA) > 1.56) demonstrate a >7-fold risk of myocardial infarction. These patients might therefore be suitable for advanced medical therapies including PCSK9 or interleukin 1-beta inhibition, with ^18^F-NaF PET used for targeting these expensive drugs to patients at greatest risk. Patients without coronary ^18^F-NaF uptake (CMA = 0) have an excellent prognosis with no myocardial infarctions observed during follow-up despite advanced coronary artery disease. In these patients with dormant coronary artery disease (a third of the population studied), further intensification of medical therapy might not be warranted, nor might they benefit on prognostic grounds from complex revascularization such as multivessel percutaneous intervention or coronary artery bypass grafting. Reprinted from Kwiecinski et al. [71], with permission from Elsevier.

While the ability to identify culprit plaques in patients with acute myocardial infarction and lesions with adverse morphology is scientifically attractive until recently, we lacked a meaningful clinical application of ^18^F-NaF coronary PET.Given that in the group of patients with established CAD prediction of adverse events has proven challenging (with current approaches based around clinical risk scores, anatomic assessments of coronary artery calcification, and the severity of obstructive coronary stenoses), we evaluated whether ^18^F-NaF could predict myocardial infarction in this population [71]. In a post hoc analysis of data collected in observational cohort studies, we showed that ^18^F-NaF PET provides a powerful independent prediction of fatal or nonfatal myocardial infarction. In 293 study participants with a high atherosclerosis burden (65% individuals had multivessel obstructive coronary artery disease, the median CT calcium score was 334 [76-804], and 81% had a history of prior revascularization), we observed 20 myocardial infarctions during almost 4 years of follow-up. On receiver operating characteristic curve analysis, the coronary microcalcification activity showed a greater area under the curve for the prediction of myocardial infarction than coronary calcium scores and clinical risk scores. To generate distinct clinical risk groups, we dichotomized the population according to their coronary ^18^F-NaF uptake. A threshold of 1.56 for CMA (derived from the receiver operating characteristic curve by the Youden index) achieved a specificity and sensitivity of 66% and 80%, respectively, for the primary endpoint. Consequently, on univariable Cox proportional regression, CMA > 1.56 emerged as a predictor of fatal or nonfatal myocardial infarction (hazard ratio: 7.30, 95% confidence interval 2.44-21.84; *p* ≤ 0.001). Importantly, these associations persisted on multivariable analysis after adjustments for sex, comorbidities, segment involvement score, number of coronary stents, multivessel coronary artery disease, coronary calcium score, and risk scores (adjusted hazard ratio of 7.1 (95% CI 2.2 to 25.1; *p* = 0.003) for the primary endpoint; Figure 4).

## 10. Conclusions and Future Directions

To date, coronary ^18^F-NaF PET has emerged as a robust tool for the assessment of active calcification processes. It has been shown to reliably identify culprit plaques, lesions with adverse morphology, and predict adverse events. Importantly the methods of image acquisition, image reconstruction, and analysis have been investigated and greatly improved over the past decade. In the near future, the outcome implications of ^18^F-NaF coronary PET will be further elucidated in the Prediction of Recurrent Events With ^18^F-Fluoride (PREFFIR) study which shall prospectively investigate the ability of ^18^F-NaF coronary PET to predict recurrent events in patients with multivessel disease and recent myocardial infarction. In regard to coronary artery disease, future studies should evaluate the potential role of ^18^F-NaF for risk stratification and guidance of therapeutic decisions in subjects with advanced atherosclerosis [23, 72]. Although to date ^18^F-NaF coronary PET research was predominantly focused on subjects with established disease, this imaging modality was also shown to identify active atherosclerotic disease across the entire spectrum of patients with subclinical, suspected, and established coronary artery disease. While in patients with advanced atherosclerosis ^18^F-NaF PET clearly outperforms clinical characteristics and scores in risk stratification, it remains to be studied whether it can add to the already-robust risk prediction in patients with the suspected disease [73]. Similar to coronary PET imaging, active calcification processes in valvular, great, and peripheral vessel disease would likely also benefit from advanced motion correction techniques and novel uptake measures. In the future, researchers should explore the potential of ^18^F-NaF PET imaging across multiple vascular beds and further improve approaches for data acquisition and analysis.
